# Antibiotic Cocktail Exacerbates Esomeprazole-Induced Intestinal Dysmotility While Ameliorating Gastric Dyspepsia in Mice

**DOI:** 10.3390/antibiotics14050442

**Published:** 2025-04-27

**Authors:** Jing-Hua Wang, Song-Yi Han, Kyungjae Lee, Uijeong Han, Si-Kyung Cho, Hojun Kim

**Affiliations:** 1 Institute of Oriental Medicine, Dongguk University, 32 Dongguk-ro, Goyang-si 10326, Gyeonggi-do, Republic of Korea; wjhdon@dongguk.edu; 2 Department of Rehabilitation Medicine of Korean Medicine, Dongguk University, 32 Dongguk-ro, Ilsandong-gu, Goyang-si 10326, Gyeonggi-do, Republic of Korea; syh2156@dgu.ac.kr; 3 College of Korean Medicine, Dongguk University, 32 Dongguk-ro, Ilsandong-gu, Goyang-si 10326, Gyeonggi-do, Republic of Korea; leeconomy@dongguk.ac.kr; 4 Department of Biological and Environmental Science, Dongguk University, 32 Dongguk-ro, Ilsandong-gu, Goyang-si 10326, Gyeonggi-do, Republic of Korea; pandasla@dgu.ac.kr (U.H.); sk.cho@dgu.ac.kr (S.-K.C.)

**Keywords:** antibiotic cocktail, esomeprazole, intestinal motility, gastric motility, gut microbiome

## Abstract

**Background/Objectives:** Esomeprazole, a proton pump inhibitor (PPI), is commonly prescribed for gastric-acid-related disorders but has been associated with impaired gastrointestinal (GI) motility with long-term use. However, the effect of concurrent antibiotic administration on this dysfunction remains unclear. Therefore, this study aimed to investigate the effects of antibiotics on esomeprazole-induced GI motility dysfunction and explore the underlying mechanisms in a mouse model. **Methods:** Male C57BL/6 mice were orally administered esomeprazole (160 mg/kg) five times per week for 4 weeks. Three days before initiating esomeprazole treatment, a broad-spectrum antibiotic cocktail (ABX) consisting of ampicillin (1 g/kg), neomycin (1 g/kg), metronidazole (1 g/kg), and vancomycin (0.5 g/kg) was provided in drinking water and maintained throughout the experimental period. Mosapride (3 mg/kg), a prokinetic agent, was used as a positive control. **Results:** Neither esomeprazole alone nor in combination with ABX affected body weight or food intake. Compared to normal controls, esomeprazole treatment significantly delayed both intestinal transit and gastric emptying. However, ABX co-administration further pronounced intestinal transit time and improved gastric motility. The potential mechanisms may involve interactions among gastric H^+^/K^+^-ATPase, CYP3A11, gastrointestinal hormones (secretin and motilin), and the gut microbiome. **Conclusions:** Long-term esomeprazole use can impair both gastric and intestinal motility, and ABX co-treatment further exacerbates intestinal transit delay while paradoxically enhancing gastric emptying. These findings highlight the critical role of the gut microbiota in esomeprazole-induced GI motility dysfunction and suggest that antibiotic use should be approached with caution, particularly when combined with PPI therapy.

## 1. Introduction

Esomeprazole, a proton pump inhibitor (PPI), is widely prescribed to treat acid-related disorders, including gastroesophageal reflux disease and peptic ulcers [1]. It acts by irreversibly inhibiting the gastric H^+^/K^+^ ATPase enzyme, thereby reducing gastric acid secretion and providing symptomatic relief [2]. Although esomeprazole is generally well tolerated, prolonged or high-dose administration may contribute to adverse effects, including impaired gastrointestinal (GI) motility, altered nutrient absorption, and gut microbiome dysbiosis [3,4,5]. Clinically, long-term PPI users frequently report symptoms such as bloating, constipation, and altered bowel habits, which suggest disturbances in normal GI motility [6,7].

To understand how esomeprazole may lead to these motility issues, it is important to consider the regulatory mechanisms of GI motility. GI motility is regulated by complex interactions among the enteric nervous system, gut hormones, and commensal microbiota [8,9]. The suppression of gastric acid secretion by esomeprazole may contribute to motility dysfunction by modulating intestinal pH, impairing digestive efficiency, and influencing the secretion of motility-regulating hormones such as motilin and secretin [10]. Several studies have shown that PPIs are associated with delayed gastric emptying and intestinal transit, although the underlying mechanisms remain only partially understood [11,12]. Emerging hypotheses suggest that these effects may involve changes in gut microbiota composition, shifts in microbial metabolites such as short-chain fatty acids (SCFAs), and impaired neuromuscular signaling [13,14].

Recent evidence underscores the critical role of the gut microbiome in modulating GI motility through complex interactions with the hormonal, neural, and immune systems [9]. Commensal microbial communities influence intestinal function by producing bioactive metabolites, engaging in receptor-mediated signaling, and interacting directly with host cells [15]. For instance, commensal bacteria produce metabolites such as bile acids that influence enteric neurons and smooth muscle activities, thereby coordinating peristalsis [16]. Disruptions in microbial composition, commonly termed dysbiosis, have been associated with motility disorders, such as irritable bowel syndrome and small intestinal bacterial overgrowth (SIBO) [17]. Notably, both antibiotics and PPIs are capable of inducing dysbiosis. Antibiotics are well known to disrupt the balance between beneficial and pathogenic bacteria [18], while esomeprazole has also been associated with microbial alterations [19]. Therefore, the concurrent administration of antibiotics and esomeprazole may have additive or even synergistic effects on gut microbial disruption, potentially leading to exacerbated GI dysmotility.

Despite increasing evidence associating both esomeprazole and antibiotics individually with GI dysmotility, their combined effects remain inadequately understood [20]. Considering the widespread co-prescription of PPIs and antibiotics in clinical settings, especially for *Helicobacter pylori* eradication therapy [21], determining whether antibiotic-induced dysbiosis aggravates PPI-related disruptions in GI motility is crucial. Therefore, addressing this knowledge gap using systematic experimental models may provide critical evidence for the underlying mechanisms and inform therapeutic strategies to mitigate adverse effects.

Accordingly, this study aimed to investigate the impact of an antibiotic cocktail (ABX) on esomeprazole-induced GI motility dysfunction and to elucidate the underlying mechanisms in a mouse model. The analysis focused on GI hormones, microbial community structure, and microbiota-derived metabolites such as SCFAs. We hypothesized that combined esomeprazole and ABX administration would result in more severe GI dysmotility due to exacerbated microbiota disruption. The findings from this study are expected to enhance our understanding of the microbiota’s role in PPI-associated motility disorders and support the development of microbiome-based therapeutic interventions.

## 2. Results

### 2.1. Long-Term Esomeprazole and Antibiotic Cocktail Administration Did Not Reduce Body Mass or Food Intake

**Figure 1:**
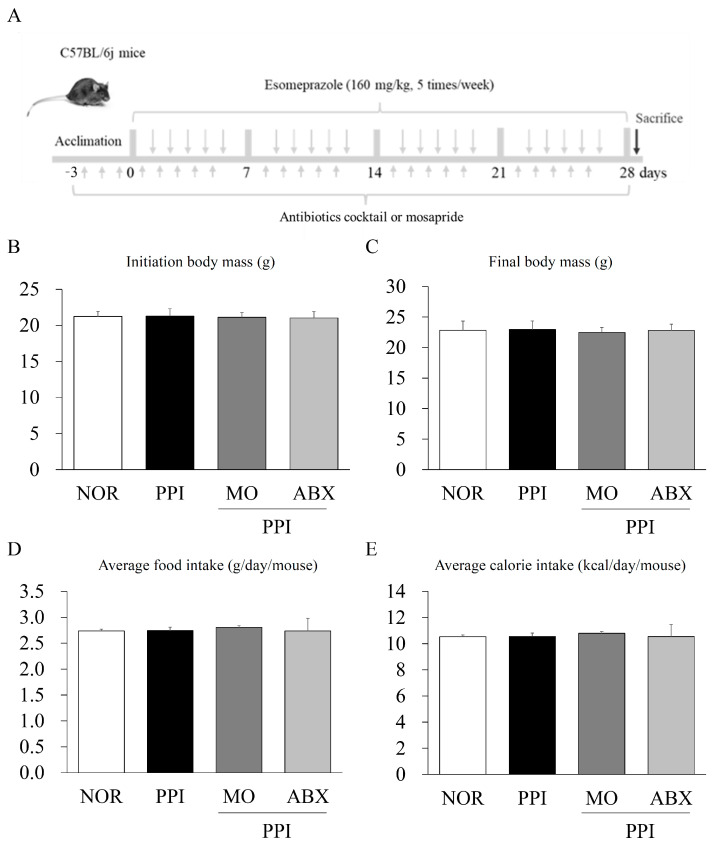
**Experimental design, average body mass, and food consumption assessment**. (**A**) Schematic representation of the experimental protocol. (**B**) Mean body weight on the initial day and (**C**) final day of the experimental period (n = 8 per group). (**D**) Mean daily food consumption and (**E**) caloric intake throughout the whole experiment. NOR: Normal, PPI (proton pump inhibitor): Esomeprazole, MO: Mosapride, antibiotic cocktail: antibiotic cocktail.

At baseline and on the 4th week, neither the PPI nor the PPI + ABX treatment significantly altered the subjects’ body weight compared to that of the normal group (*p* > 0.05, Figure 1B,C). Throughout the experimental period, neither treatment led to a significant change in average food intake or caloric intake relative to the normal group (*p* > 0.05, Figure 1D,E).

### 2.2. Antibiotic Cocktail Alleviated the Proton-Pump-Inhibitor-Induced Inhibition of Gastric Motility

**Figure 2:**
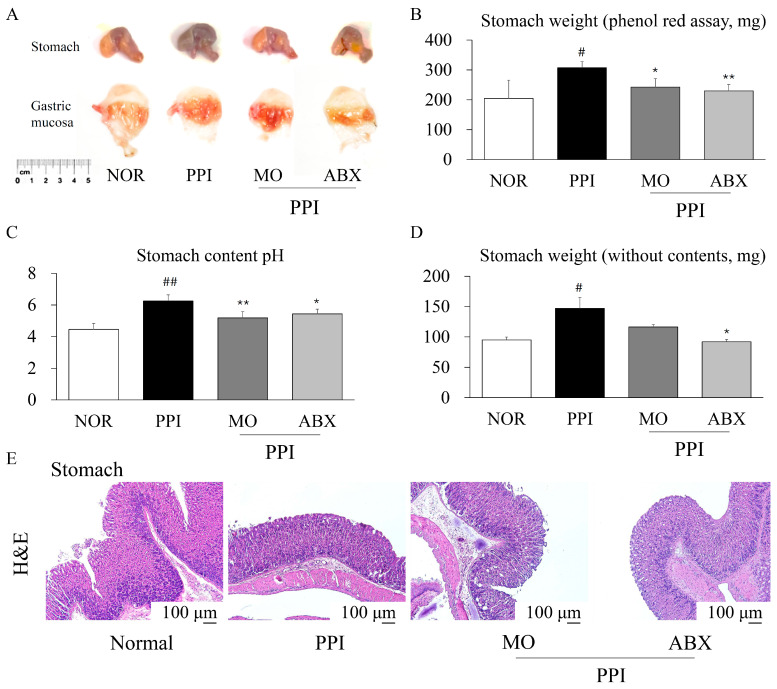
**The ABX ameliorated PPI-induced inhibition of gastric emptying**. (**A**) Morphology of the stomach and gastric mucosa after intragastric administration of phenol red. (**B**) Stomach weight containing phenol red, (**C**) pH of stomach content, and (**D**) stomach weight without content were compared on the final experimental day. (**E**) Stomach tissue was stained by hematoxylin and eosin (n = 4 per group). NOR: Normal, PPI: Esomeprazole, MO: Mosapride, ABX: Antibiotic cocktail. ^##^ *p* < 0.01 and ^#^ *p* < 0.05, compared to the normal group; * *p* < 0.05, ** *p* < 0.01, compared to the PPI group.

The long-term PPI treatment significantly increased both the phenol-red-containing stomach weight and stomach weight without content compared to normal conditions (*p* < 0.05, Figure 2A–D). However, the ABX combination treatment did not increase stomach weight, with or without content (Figure 2D). Gastric pH was significantly elevated in the PPI group compared to the normal group (*p* < 0.01). Both the mosapride (MO) and ABX treatments increased gastric acidity, as reflected by a significant reduction in pH compared to the PPI group (*p* < 0.01 or *p* < 0.05, Figure 2C).

Histological analysis with hematoxylin and eosin (H&E) staining revealed mucosal thickening and glandular hyperplasia in the PPI-treated groups. In contrast, the ABX group maintained a relatively preserved gastric mucosal architecture, similar to that of the normal group (Figure 2E).

### 2.3. Antibiotic Cocktail Exacerbated the Proton-Pump-Inhibitor-Induced Inhibition of Intestinal Motility

**Figure 3:**
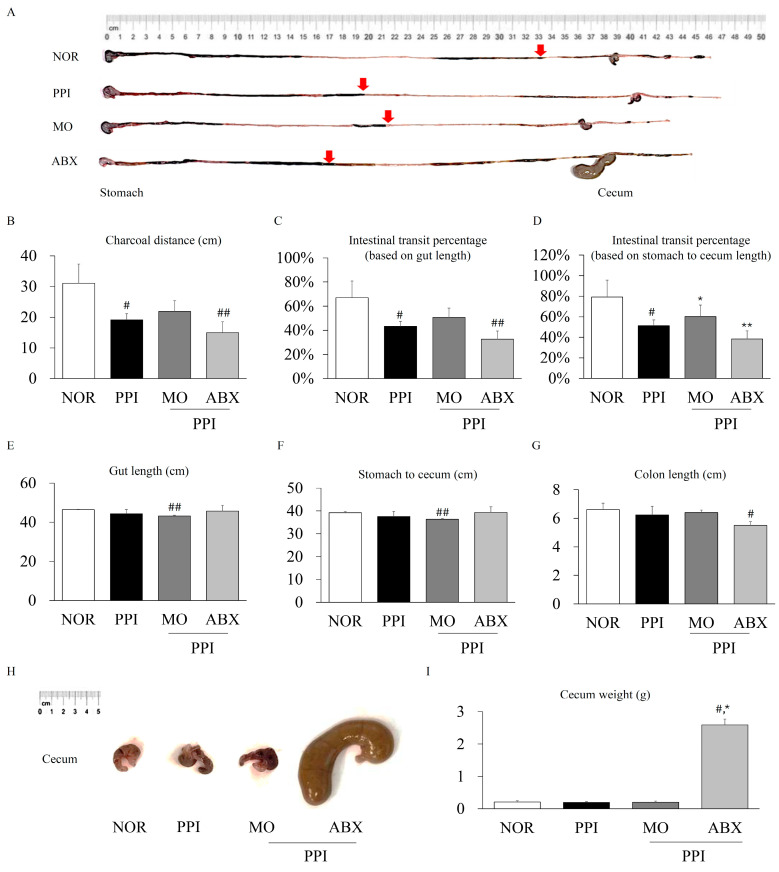
**ABX exacerbates esomeprazole-induced inhibition of intestinal transit**. (**A**) Morphology of the intestine after intragastric administration of charcoal. (**B**) Charcoal transit distance, (**C**) intestinal transit rate (divided by total gut length), and (**D**) intestinal transit rate (divided by stomach to cecum length) were compared individually. (**E**) Gut length, (**F**) stomach to cecum length, and (**G**) colon length were compared. Cecum (**H**) morphology and (**I**) weight were compared on the last experimental day. NOR: Normal, PPI: Esomeprazole, MO: Mosapride, ABX: Antibiotic cocktail. ^##^ *p* < 0.01 and ^#^ *p* < 0.05, compared to the normal group; * *p* < 0.05, ** *p* < 0.01, compared to the PPI group.

The long-term PPI treatment significantly decreased the charcoal transit distance and overall intestinal transit compared to normal conditions (*p* < 0.05, Figure 3A–D); however, it had no significant effect on gut length or cecum weight (Figure 3E–I). However, the ABX combination treatment led to a pronounced reduction in the charcoal distance and intestinal transit. Interestingly, the ABX combination treatment dramatically increased the cecum weight (*p* < 0.05) and significantly reduced the colon length compared to those under normal conditions (*p* < 0.05, Figure 3I).

### 2.4. Antibiotic Cocktail Deteriorated the Proton-Pump-Inhibitor-Induced Injury of Intestinal Integrity

**Figure 4:**
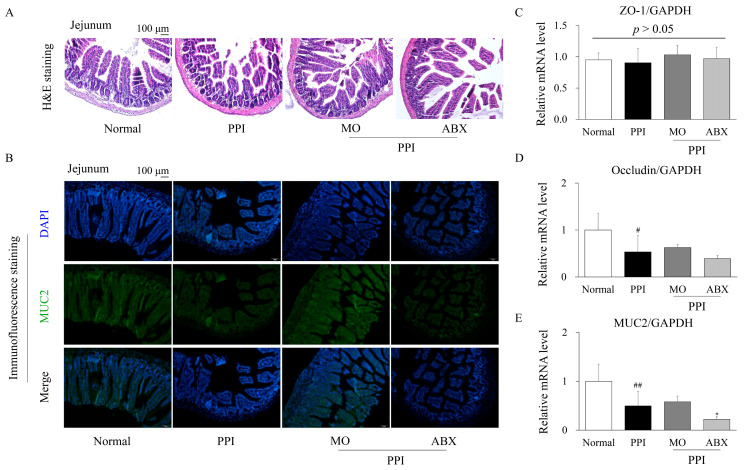
**The ABX cocktail changed the histopathological structure in the intestine**. (**A**) Representative hematoxylin and eosin (H&E) staining images of jejunal sections showing villus morphology. (**B**) Immunofluorescence staining for MUC2 (green) and nuclei (DAPI, blue) in jejunal tissues. Quantitative real-time PCR analysis of tight junction and mucus-related genes: (**C**) ZO-1, (**D**) Occludin, and (**E**) MUC2 mRNA expression levels normalized to Glyceraldehyde 3-phosphate dehydrogenase (GAPDH). Data are presented as mean ± SD. PPI: Esomeprazole, MO: Mosapride, ABX: Antibiotic cocktail. ^#^ *p* < 0.05, ^##^ *p* < 0.01 vs. normal group; * *p* < 0.05 vs. PPI group.

To evaluate the histopathological alterations in the jejunum induced by PPI treatment and the ABX combination, H&E staining and immunofluorescence staining for MUC2 were performed. H&E staining revealed that compared to the normal group, PPI administration disrupted the intestinal villus structure, leading to villus shortening and broadening (Figure 4A). The ABX group exhibited further exacerbation of villous atrophy and reduced crypt depth, indicating significant impairment of the intestinal barrier.

Immunofluorescence staining for MUC2 revealed a noticeable reduction in MUC2 expression in the PPI-treated group compared to the normal group, suggesting depletion of the mucosal barrier (Figure 4B). The ABX group revealed an even greater decline in MUC2 fluorescence intensity, supporting the hypothesis that microbiota depletion by ABX exacerbates mucus layer impairment.

Real-time quantitative PCR (qPCR) analysis of tight junction proteins and mucus-related gene expression further supported these findings. No significant difference was observed in the mRNA expression of ZO-1 among the groups (*p* > 0.05; Figure 4C). However, occludin expression was significantly downregulated in the PPI group compared to the normal group (*p* < 0.05), with no further reduction in the ABX combination group (Figure 4D). Notably, MUC2 mRNA expression was significantly lower in the PPI group (*p* < 0.01) than in the ABX group (*p* < 0.05), indicating pronounced impairment of the intestinal mucus barrier (Figure 4E).

### 2.5. Antibiotic Cocktail Impacted Proton Pump Inhibitor Metabolic Enzymes, GI Hormones, and Inflammatory Markers

**Figure 5:**
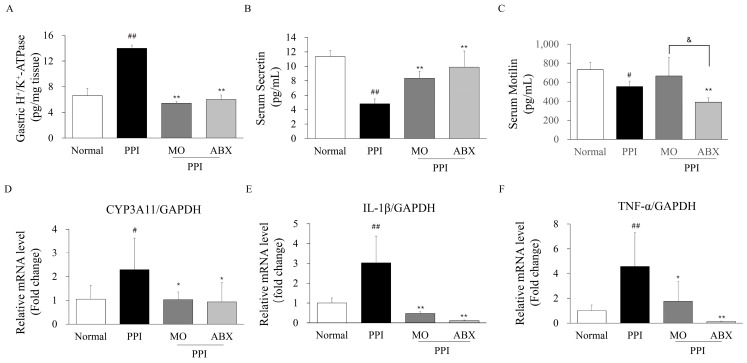
**ABX potentially altered PPI metabolic enzymes, GI hormones, and inflammatory cytokines**. (**A**) Gastric H^+^/K^+^-ATPase levels were measured using an enzyme-linked immunosorbent assay (ELISA) kit. Serum (**B**) secretin and (**C**) motilin levels were evaluated using an ELISA kit. Gene expression of pro-inflammatory cytokines (**D**) IL-1β, (**E**) TNF-α, and the key drug metabolic enzyme (**F**) CYP3A11 in intestinal tissues, normalized to GAPDH and presented as fold change relative to the normal group. PPI: Esomeprazole, MO: Mosapride, ABX: Antibiotic cocktail. * *p* < 0.05, ** *p* < 0.01 vs. normal group; ^#^ *p* < 0.05, ^##^ *p* < 0.01 vs. PPI group; ^&^ *p* < 0.05 between indicated groups.

As a proton pump, gastric H^+^/K^+^-ATPase levels were significantly higher in the PPI group than in the normal group (*p* < 0.01; Figure 5A). However, gastric H+/K+-ATPase levels did not increase in the ABX and MO combination groups.

Serum secretin levels were significantly reduced in the PPI group (*p* < 0.01), whereas both the MO and ABX combination treatments significantly restored secretin levels (*p* < 0.01, Figure 5B). In addition, serum motilin levels were significantly lower in the PPI group than in the normal group (*p* < 0.05; Figure 5C). Notably, the ABX combination treatment further suppressed motilin levels compared to the PPI group (*p* < 0.05), indicating a synergistic inhibitory effect of ABX on motilin secretion.

The expression of CYP3A11, a key enzyme in PPI metabolism, was significantly increased in the PPI group (*p* < 0.05, Figure 5D). However, both the MO and ABX combination treatments resulted in a significant decrease in the gene expression of CYP3A11 compared to the PPI group (*p* < 0.05). Furthermore, the gene expression of inflammatory cytokines was examined using qPCR. The mRNA levels of IL-1β and TNF-α were significantly elevated in the PPI group compared to the normal group (*p* < 0.01, Figure 5E,F). However, both the MO and ABX combination treatments significantly reduced IL-1β (*p* < 0.01) and TNF-α expression (*p* < 0.05 or *p* < 0.01), indicating an attenuation of PPI-induced inflammation.

### 2.6. Antibiotic Cocktail Further Reduced Certain Fecal Short-Chain Fatty Acids Levels and Related Receptor

**Figure 6:**
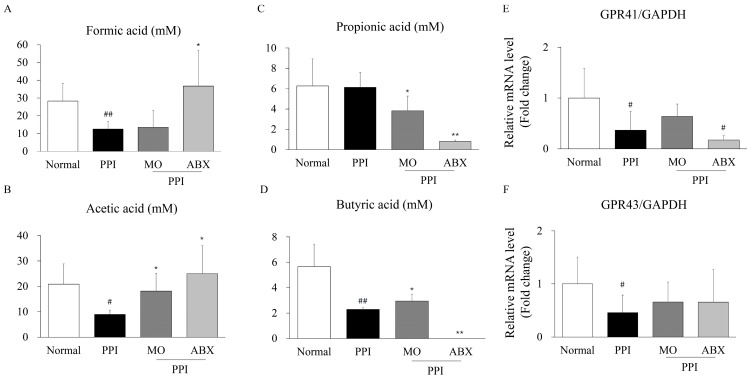
**ABX further suppressed low levels of fecal SCFA and its receptor**. Fecal concentrations of formic acid (**A**), acetic acid (**B**), propionic acid (**C**), and butyric acid (**D**) were examined using high-performance liquid chromatography. Gene expression of SCFA receptors GPR41 (**E**) and GPR43 (**F**) in intestinal tissues was determined by qPCR. Gene expression was normalized to GAPDH and presented as fold change relative to the normal group. PPI: Esomeprazole, MO: Mosapride, ABX: Antibiotic cocktail. * *p* < 0.05, ** *p* < 0.01 vs. normal group; ^#^ *p* < 0.05, ^##^ *p* < 0.01 vs. PPI group.

Fecal formic acid and acetic acid levels were significantly lower in the PPI group than in the normal group (Figure 6A,B, *p* < 0.01 or *p* < 0.05). However, the ABX group revealed a significant increase in these levels compared to the PPI group (*p* < 0.05).

Fecal propionic acid levels in the PPI group were unchanged compared to the normal group but were significantly reduced in the ABX group compared to the PPI group (*p* < 0.05, Figure 6C). Interestingly, fecal butyric acid levels were significantly lower in the PPI group than in the normal group (*p* < 0.01; Figure 6D), with the ABX group exhibiting a more pronounced depletion of butyric acid (*p* < 0.01).

Additionally, the expression levels of two key SCFA receptor genes were assessed. GPR41 mRNA expression was significantly downregulated in the PPI group (*p* < 0.05; Figure 6E) and revealed a further decline in the ABX group (*p* < 0.05). Similarly, GPR43 expression was significantly reduced in the PPI group (*p* < 0.05; Figure 6F), with no recovery observed in either the MO or ABX groups.

### 2.7. Antibiotic Cocktail Significantly Reduced Gut Microbiome Diversity but May Increase the Absolute Bacterial Load

**Figure 7:**
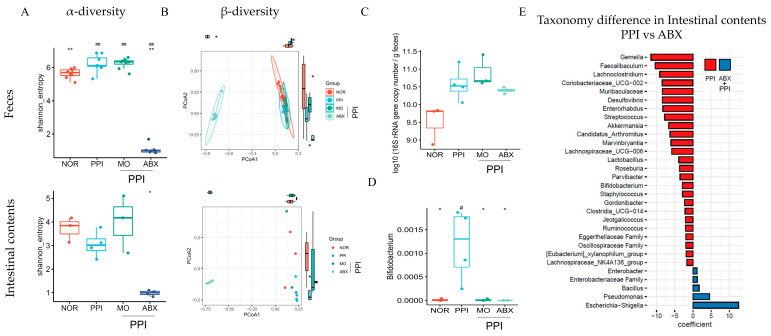
**Alteration of gut microbiome diversity and composition**. (**A**) α-diversity (Shannon index) of fecal (n = 6) and intestinal (n = 4) content microbiota. (**B**) β-diversity (PCoA analysis based on Bray–Curtis distance) of fecal and intestinal content microbiota. (**C**) Total fecal bacterial abundance measured by 16S rRNA gene copy number. (**D**) Relative abundance of Bifidobacterium in small intestinal contents. (**E**) Taxonomic differences in intestinal contents between PPI and ABX groups, showing significantly enriched or depleted genera (red bars indicate enrichment in ABX, and blue bars indicate enrichment in PPI). NOR: Normal, PPI: Esomeprazole, MO: Mosapride, ABX: Antibiotic cocktail. * *p* < 0.05, ** *p* < 0.01 compared to NOR; ^#^ *p* < 0.05, ^##^ *p* < 0.01 compared to PPI.

As presented in Figure 7A, the Shannon index of the fecal microbiota was drastically reduced in the ABX group compared to that in the normal and PPI group (*p* < 0.01). A similar trend was observed in the intestinal contents.

A β-diversity analysis revealed the distinct clustering of microbial communities among groups, with the ABX group revealing clear separation from the normal (NOR), PPI, and MO groups (Figure 7B). The total fecal bacterial abundance (Figure 7C), quantified by the total 16S rRNA gene copy number, was significantly higher in the PPI and PPI+ABX group than in the NOR group (*p* < 0.01).

Compared with the normal group, the relative abundance of Bifidobacterium (Figure 7D) was significantly higher in the PPI group (*p* < 0.01). However, the ABX combination treatment resulted in a relatively low level of Bifidobacterium in the small intestine. Taxonomic comparisons between the PPI and ABX groups revealed alterations in specific bacterial genera. The ABX combination treatment led to a marked reduction in Gemella, Faecalibaculum, and Lachnoclostridium, while Pseudomonas and Escherichia-Shigella were enriched compared to the PPI group (Figure 7E).

## 3. Discussion

Esomeprazole, a widely prescribed PPI, is a first-line treatment for many acid-related disorders due to its potent efficacy and favorable tolerability profile [22,23]. However, accumulating evidence suggests that long-term esomeprazole administration is associated with a range of adverse gastrointestinal effects [7]. Prolonged acid suppression may predispose patients to gut microbiome dysbiosis, increasing the risk of intestinal infections, nutrient malabsorption, and GI motility disorders [7,24]. Clinically, antibiotics are frequently co-administered with esomeprazole, particularly for the treatment of Helicobacter pylori infections and gastric ulcers [25,26]. Nevertheless, whether combination therapy with antibiotics and esomeprazole increases the risk of gastrointestinal motility disorders and, if so, through what mechanisms remains unclear. Therefore, we compared the results for gastrointestinal motility after combination therapy and esomeprazole monotherapy and investigated their potential underlying mechanisms in a long-term mouse model.

First, we did not observe significant changes in appetite or body mass following long-term high-dose esomeprazole administration. However, both gastric emptying and intestinal transit were significantly delayed by esomeprazole, as indicated by charcoal and phenol red assays. A conceivable explanation is that while esomeprazole does not directly or noticeably affect appetite and daily metabolism, long-term acid suppression alters gastrointestinal physiology by reducing gastric acid secretion, which disrupts the normal feedback mechanisms regulating gastrointestinal motility.

Gastric acid plays a critical role not only in digestion but also in stimulating gastric emptying and intestinal peristalsis [27]. As expected, our study confirmed that esomeprazole significantly increased gastric pH in mice. The resulting hypochlorhydria likely plays an essential role in delaying gastric emptying and reducing intestinal transit [28]. The reduction in gastric pH and enhancement of gastric emptying observed with ABX co-administration indicated that ABX may ameliorate esomeprazole-induced gastric motility impairment. Moreover, the morphology of the gastric mucosa and stomach weight also indicated that long-term esomeprazole administration led to gastric wall thickening, which might be associated with chronic mucosal hyperplasia induced by gastric inflammation and microbiota dysbiosis [29,30]. In contrast, ABX co-administration significantly reduced bacterial overgrowth and inflammatory responses, normalizing the gastric wall thickness and improving gastric emptying. In addition to their effects on gastrointestinal motility, both MO and ABX were associated with significant alterations in gut morphology, including changes in intestinal length and cecum weight. MO, a 5-HT_4_ receptor agonist known to enhance gastrointestinal peristalsis, appeared to reduce the gut transit time and shorten the intestinal length, likely through increased mechanical stimulation and the adaptive remodeling of the intestinal wall. In contrast, the ABX treatment markedly increased the cecum weight and shortened the gut length, most likely due to the depletion of commensal gut microbiota. Microbiota depletion has been reported to cause cecal enlargement through fluid accumulation, the retention of undigested contents, and impaired microbial fermentation [31]. These morphological changes reflected the profound impact of microbial disruption on gut physiology and highlight the intricate interplay between pharmacological interventions and host–microbiota interactions.

Generally, alterations in gastric acidity can influence the gut microbiota by reducing the natural barrier of the stomach against ingested microbes, potentially promoting microbial overgrowth in the upper GI tract and shifting in microbial composition further downstream [32]. Hypochlorhydria increases the risk of SIBO, which can generate excessive gas and reduce the levels of certain bacterial metabolites, such as SCFAs, thereby impairing the neuromuscular function of the GI tract [13,33].

In our study, the esomeprazole treatment significantly increased the total bacterial abundance in the intestine, as evidenced by higher 16S rRNA gene copy numbers. This finding suggests that esomeprazole-induced hypochlorhydria, which is characterized by reduced gastric acidity and the increased availability of undigested carbohydrates, may create a favorable environment for bacterial overgrowth, particularly promoting Bifidobacterium proliferation. However, broad-spectrum antibiotics typically reduce the overall diversity of the gut microbiota, leading to the dominance of a limited number of antibiotic-resistant or antibiotic-resilient bacterial species [34]. ABX co-administration did not significantly alter the total bacterial load in the intestinal contents compared to esomeprazole but dramatically reduced the intestinal microbial species diversity. Upon comparison, several bacterial genera, including Pseudomonas and Escherichia-Shigella, were significantly increased following ABX co-administration. Under the conditions of a microbiome imbalance, such as prolonged antibiotic administration, Pseudomonas can act as an opportunistic pathogen that impairs intestinal function [35]. Shiga toxin, a major Shigella virulence factor, initially triggers intestinal hypermotility by inducing inflammation and epithelial damage [36]. However, chronic toxin-induced neuronal injury and muscle layer edema can suppress gut motility, potentially leading to hypomotility or even paralysis [37,38]. The markedly enlarged cecum after ABX co-administration indicated intestinal dysfunction involving motility impairment and gut microbiota disruption.

Immunofluorescence and pathological observations confirmed that ABX exacerbated esomeprazole-induced intestinal barrier dysfunction. Interestingly, while the combined esomeprazole and ABX treatment significantly reduced occludin expression, ZO-1 levels remained relatively stable. This differential response can be attributed to their distinct functional roles in the tight junction complexes. Occludin, a transmembrane protein, is highly sensitive to microbial dysbiosis-induced damage, leading to its rapid downregulation and compromised barrier function [39]. However, ZO-1 mainly serves as a structural adaptor linking tight junction proteins to the actin cytoskeleton, making its expression more resistant to environmental stress unless severe epithelial disruption occurs [40]. These findings indicate that gut microbiota dysbiosis induced by antibiotics can exacerbate esomeprazole-induced intestinal dysmotility, accompanied by increased colonization by opportunistic pathogens. The observed gut microbiota alterations appear to impair intestinal motility rather than gastric motility, possibly due to the greater ecological complexity of the intestinal microbiome compared to that of the stomach.

Additionally, butyric acid, a key SCFA produced by the gut commensal microbiome, serves as the primary energy source for colonic epithelial cells, maintaining intestinal barrier integrity, reducing inflammation, and promoting GI motility [41,42]. By binding to its receptors, GPR41 and GPR43, it activates the enteric nervous system and modulates neural signaling pathways, which, in turn influence smooth muscle contraction and relaxation, thus increasing intestinal motility [43,44]. In this study, esomeprazole-induced gut microbiome dysbiosis led to a significant reduction in fecal SCFA levels, particularly butyric acid, along with the downregulation of SCFA receptor gene expression. This effect is likely attributable to the loss of butyrate-producing bacteria and disruption of cross-feeding mechanisms. The co-administration of ABX further decreased fecal butyric and propionic acid levels, whereas formic and acetic acid levels remained relatively unchanged. This was consistent with a further reduction in intestinal GPR41 expression, which aggravated the impairment of intestinal motility. Notably, GPR43 expression was not suppressed further by antibiotic treatment, suggesting that GPR41 is more sensitive to butyrate depletion and enteric neural disruption. In addition, ABX co-treatment reduced the abundance of known butyrate-producing bacteria [45], such as Faecalibacterium and Roseburia, compared to PPI treatment alone. Based on these findings, we propose that antibiotic co-treatment exacerbates esomeprazole-induced intestinal transit delay by further reducing butyrate production, leading to the suppression of GPR41-mediated signaling and consequent impairment of intestinal motility.

Notably, esomeprazole is a prodrug that becomes activated in the acidic environment of gastric parietal cells, where it is converted into a reactive sulfenamide compound [46,47]. This active metabolite covalently binds to sulfhydryl groups on the H^+^/K^+^-ATPase enzyme, inducing irreversible conformational changes that render the enzyme nonfunctional [48]. Importantly, esomeprazole inhibits the activity of H^+^/K^+^-ATPase rather than reducing its quantity. Therefore, we observed a compensatory upregulation of H^+^/K^+^-ATPase and gene expression of intestinal CYP3A11, a critical enzyme for esomeprazole metabolism, in the esomeprazole-treated group. However, the ABX co-treatment notably normalized the levels of H^+^/K^+^-ATPase and intestinal CYP3A11 expression, potentially mitigating microbiota dysbiosis, reducing intestinal inflammation, and subsequently decreasing the compensatory metabolic burden associated with chronic esomeprazole administration.

Eventually, due to the use of three distinct experimental sets, the correlation analysis could not be comprehensively conducted. It should be noted that the esomeprazole dose used in this study exceeds typical clinical exposures. While this mid-range dose was selected based on previous rodent studies to ensure the consistent induction of GI dysfunction, we acknowledge its limitations in terms of translational relevance. Future studies incorporating a dose–response design are warranted to better define threshold effects and evaluate the clinical applicability of our findings. In addition, we acknowledge that this experimental design primarily addressed the long-term effects of dysbiosis on gastrointestinal motility by antibiotics, rather than distinguishing between immediate and delayed responses. Moreover, potential microbiota-targeted interventions, such as the use of prebiotics, probiotics, and fecal microbiota transplantation (FMT), have shown promise in restoring microbial balance and improving gastrointestinal motility [49,50]. These strategies may offer therapeutic value in mitigating PPI-induced dysbiosis and its associated functional impairments. Further research is warranted to investigate the efficacy and underlying mechanisms of these interventions in the context of PPI-associated gastrointestinal dysfunction.

## 4. Materials and Methods

### 4.1. Animals and Experimental Design

Forty-eight male C57BL/6J mice (6 weeks old) were procured from Daehan Biolink (Eumsung, Republic of Korea). Following a 7-day acclimation period under controlled environmental conditions (temperature: 22 ± 2 °C, relative humidity: 40 ± 10%, and a 12 h light/dark cycle), the mice were provided ad libitum access to a standard chow diet and water. The mice were randomly allocated to four experimental groups (n = 12 per group): NOR control, PPI, MO, and ABX. Each group was further categorized into three subgroups of four mice each: one for the gastric emptying test, one for the intestinal motility test, and one for mechanism evaluation.

Except for the normal control group, all mice received esomeprazole (160 mg/kg) via oral gavage five times per week for 4 weeks (Figure 1A). To minimize the risk of toxicity, the esomeprazole dose was determined according to prior experimental data in mice [51]. Three days prior to the initiation of experimental treatments, all mice, except those in the normal group, were administered an ABX consisting of ampicillin (1 g/kg body weight), neomycin (1 g/kg body weight), metronidazole (1 g/kg body weight), and vancomycin (0.5 g/kg body weight) via oral gavage according to a previous study [52]. This antibiotic regime was continued daily throughout the 31-day experimental period. As a positive control, mice in the MO group were treated with mosapride (3 mg/kg) five times per week for 4 weeks. At the end of the experiment, all mice were euthanized, and blood samples were collected from the inferior vena cava for serum analysis. Stomach and small intestine tissues were excised and preserved under varying conditions: samples were either snap-frozen and stored at −80 °C, fixed in 4% paraformaldehyde, or stabilized in RNAlater solution (Invitrogen, Carlsbad, CA, USA) for subsequent molecular analyses. The pH of the gastric contents was measured using a pH indicator strip (pH range: 3.4–6.4; Toyo Roshi Kaisha, Tokyo, Japan).

The experimental protocol involving animals was reviewed and approved by the Institutional Animal Ethics Committee of Dongguk University (approval no. IACUC-2024-04262), and the study was conducted in strict accordance with the guidelines established by the United States National Institutes of Health (NIH) for the humane care and use of laboratory animals. To minimize distress during euthanasia, all animals were anesthetized with a balanced combination of Zoletil (tiletamine–zolazepam; Virbac, Carros, France) and Rompun (xylazine hydrochloride; Bayer, Leverkusen, Germany), administered in equal proportions. The protocol followed established veterinary standards to optimize animal welfare.

### 4.2. Charcoal Assay for Intestinal Motility Assessment

After a 20 h fasting period, each mouse was orally administered 200 μL of a 5% charcoal suspension prepared in 0.5% carboxymethylcellulose (CMC) as a non-absorbable marker. After 30 min, the mice were euthanized, and their gastrointestinal tracts were harvested. The progression of charcoal through the intestine was assessed by measuring the distance traveled, and the intestinal transit rate was calculated using the following formula: intestinal transit rate (%) = (charcoal distance/gut length) × 100%. For this calculation, total gut length was defined as the distance from the stomach to the cecum.

### 4.3. Phenol Red Assay for Gastric Emptying Evaluation

On the final experimental day, following a 20 h fasting period, mice were orally administered 200 μL of a 5% charcoal suspension in 0.5% CMC to assess intestinal transit. Gastric emptying was evaluated using a nonabsorbable dye, which was administered orally. After 30 min, the mice were sacrificed under anesthesia, after which their stomachs were excised. Gastric emptying was assessed based on gross morphological findings, gastric weight, and measurements of the stomach and associated areas.

### 4.4. Histopathological Analysis Using Hematoxylin and Eosin and Immunofluorescence Staining

The stomach and jejunal tissues were fixed in 4% paraformaldehyde, embedded in paraffin, and sectioned into 4 μm thick slices for histological analysis. After drying, liver tissue sections were deparaffinized, rehydrated, stained with H&E, dehydrated, and mounted using Clarion™ mounting medium. For immunofluorescence staining, paraffin-embedded jejunal tissues were sectioned at 4 μm. Following deparaffinization and rehydration, antigen retrieval was performed using heat-induced methods, and non-specific binding was blocked with 2% goat serum. The sections were then incubated with a 1:100 dilution of MUC2 antibody (996/1, conjugated to DyLight 488). Finally, the slides were counterstained with 4′,6-diamidino-2-phenylindole and mounted using an antifade medium. Fluorescence images were captured using an optical microscope (BX61; Olympus, Tokyo, Japan), and fluorescence intensity was quantified using ImageJ software (version 1.54g, 18 October 2023, NIH, Bethesda, MD, USA).

### 4.5. Measurement of Gastrointestinal Hormones in Serum by Enzyme-Linked Immunosorbent Assay

Mice gastric tissue (100 mg) was homogenized in 1 mL of radioimmunoprecipitation assay buffer at 4 °C. The homogenate was then centrifuged at 5000× *g* for 5 min, and 100 μL of the resulting supernatant was used to quantify the concentrations of H^+^/K^+^ ATPase (catalog no. EKU02630) using enzyme-linked immunosorbent assay (ELISA) kits following the manufacturer’s instructions (BIOMATIK, Kitchener, ON, Canada). Mouse serum motilin (catalog no. EKU09404) and secretin (Catalog No. EKU077227) were determined using ELISA kits, following the manufacturer’s instructions (BIOMATIK, ON, Canada).

### 4.6. Quantification of Fecal Short-Chain Fatty Acids

Fecal filtrates were transferred into 1.5 mL screw-cap vials fitted with glass inserts for high-performance liquid chromatography (HPLC) analysis [53]. The concentrations of lactate, formate, acetate, propionate, and butyrate (expressed as mM/g of mouse feces) were determined using a Shimadzu HPLC system (Shimadzu Corporation, Kyoto, Japan) equipped with an LC-20AD binary pump, SIL-20A autosampler, and SPD-20A UV/Vis detector. Chromatographic separation was performed using an Aminex HPX-87H column (300 mm × 7.8 mm, 9 µm particle size; Catalog Number: #1250140; Bio-Rad Laboratories, Hercules, CA, USA) maintained at 50 °C, with a constant mobile-phase flow rate of 0.6 mL/min. The system operated in isocratic mode with 0.005 M sulfuric acid as the mobile phase, and analyte detection was performed at 210 nm. The injection volume was set at 10 µL, with a total elution time of 40 min per sample. Calibration standards for SCFAs were prepared across a concentration range of 0.1–200 mM, and corresponding calibration curves were constructed, yielding correlation coefficients exceeding 0.999, ensuring robust linearity and accuracy in quantification.

### 4.7. Real-Time Quantitative PCR

Total RNA was extracted from frozen mouse small intestine tissues using TRIzol reagent (Invitrogen, Carlsbad, CA, USA) according to the manufacturer’s instructions. Complementary DNA (cDNA) was synthesized from the isolated RNA using AccuPower RT PreMix (BIONEER, Daejeon, Republic of Korea), which contained Moloney Murine Leukemia Virus (M-MLV) reverse transcriptase, reaction buffer, and RNase inhibitor. Real-time qPCR was performed to assess the expression of the 10 target genes, with glyceraldehyde 3-phosphate dehydrogenase (GAPDH) serving as the endogenous housekeeping gene for normalization. Amplification conditions followed previously established protocols, and primer sequences and detailed experimental parameters are provided in Table S1 [54,55,56,57,58]. qPCR analysis was conducted using the LightCycler^®^ 96 System (Roche, Basel, Switzerland). Relative gene expression levels were quantified using the 2^−ΔCt^ method and normalized to GAPDH expression to account for variations in cDNA input.

### 4.8. Fecal and Intestinal Contents 16S rRNA Gene Sequencing

Total DNA was extracted from fecal and intestinal samples using the QIAamp PowerFecal Pro DNA Kit (QIAGEN, Hilden, Germany). A library of the hypervariable V3–V4 regions of the 16S rRNA gene was constructed following the 16S Metagenomic Sequencing Library Preparation Illumina protocol (Part #15044223 Rev. B, Illumina, San Diego, CA, USA). Libraries were sequenced on the Illumina platform. 16S rRNA gene sequencing data were analyzed using QIIME2 software (version 2023.9) [59]. The raw sequence data were trimmed and quality-controlled using FASTP (version 0.23.2), and primer/adapter sequences were removed using Cutadapt (version 4.4). The optional truncation parameters for DADA2 were optimized using Figaro (version 1.2.0). Denoising, dereplication, chimera removal, and amplicon sequence variant (ASV) table generation were performed using the DADA2 plugin within the QIIME2 framework. Amplicon sequence variant feature tables were generated using the DADA2 pipeline [60] and taxonomic assignments were performed based on SILVA (version 138) 99% 16S rRNA databases [61]. Differential abundance analysis between groups was performed using MaAsLin2 (q < 0.05, FDR threshold) [62].

### 4.9. Statistical Analysis

All data are expressed as means ± standard deviation. Statistical differences between experimental groups were evaluated using one-way analysis of variance, followed by the least significant difference post hoc test to determine group comparisons. A *p*-value of less than 0.05 was considered statistically significant. All analyses were performed using ImageJ (version 17.0, Chicago, IL, USA) to ensure robustness and reproducible results.

## 5. Conclusions

Taken together, our findings revealed that long-term esomeprazole administration impairs gastrointestinal motility by inducing hypochlorhydria, gut microbiota dysbiosis, and reduced butyrate production. Notably, antibiotic co-treatment further exacerbated intestinal motility impairment by aggravating dysbiosis, reducing butyric acid levels, and downregulating GPR41 expression. In contrast, antibiotics partially ameliorated esomeprazole-induced delayed gastric emptying, likely by mitigating prolonged acid suppression. These findings revealed the distinct, region-specific effects of microbiota alterations on gastric and intestinal motility. Therefore, the clinical use of esomeprazole in combination with antibiotics should be approached cautiously, considering the potential risk of exacerbating intestinal dysmotility due to microbiota-driven metabolic disruption.

## Data Availability

The original contributions presented in this study are included in the article/Supplementary Material. Further inquiries can be directed to the corresponding author.

## Supplementary Materials

The following supporting information can be downloaded at: https://www.mdpi.com/article/10.3390/antibiotics14050442/s1; Table S1: Information on qPCR Primers for Mouse.

## Abbreviations

The following abbreviations are used in this manuscript:

ABX	Antibiotic cocktail
ANOVA	Analysis of variance
CMC	Carboxymethylcellulose
CYP3A11	Cytochrome P450, family 3, subfamily a, polypeptide 11
ELISA	Enzyme-linked immunosorbent assay
FMT	Fecal microbiota transplantation
GAPDH	Glyceraldehyde 3-phosphate dehydrogenase
GI	Gastrointestinal
H&E	Hematoxylin and eosin
HPLC	High-performance liquid chromatography
PPI	Proton pump inhibitor
SCFAs	Short-chain fatty acids
SIBO	Small intestinal bacterial overgrowth
