# Supplementary material for: Antibiotic Cocktail Exacerbates Esomeprazole-Induced Intestinal Dysmotility While Ameliorating Gastric Dyspepsia in Mice
Source: Antibiotics (Basel). 2025 Apr 27;14(5):442. doi: 10.3390/antibiotics14050442 (PMC12108370; doi:10.3390/antibiotics14050442)
Supplement: Supplementary file 1 [file antibiotics-14-00442-s001.zip › antibiotics-3579086-supplementary.pdf]

Table S1. Information on qPCR Primers for Mouse

Gene ID	Sequence (5'~ 3')	Product size (bp)	AT (℃)	PrimerBank ID (Ref)
GAPDH	F: AGGTCGGTGTGAACGGATTTG<br>R: TGTAGACCATGTAGTTGAGGTCA	123	57	6679937a1
ZO-1	F: ACAAACAGCCCTACCAACC<br>R: CCATCCTCATCTTCATCTTCTTC	185	58	doi: 10.3390/genes1301 0151 [54]
Occludin	F: TTGAAAGTCCACCTCCTTACAGA<br>R: CCGGATAAAAAGAGTACGCTGG	129	56	6679162a1
MUC2	F: TCCAGGTCTCGACATTAGCAG<br>R: GTGCTGAGAGTTTGCGTGTCT	156	57	26351711a1
GPR41	F: CATGTGGTGGGCTATGTC<br>R: TGAGTCCAAGGCACACAAG	176	57	doi:10.7150/thno.56 598 [55]
GPR43	F: ACCATCGTCATCATCGTTCA<br>R: ACGAAGCGCCAATAACAGA	185	57	doi:10.7150/thno.56 598 [55]
CYP3A11	F: GCCATTTTTAGGCACTGTGCTGA<br>R: TGTGACAGCAAGGAGAGGCGT	124	58	doi: 10.1002/prp2.82 [56]
TNF-α	F: CGGGCAGGTCTACTTTGGAG<br>R: ACCCTGAGCCATAATCCCCT	166	60	doi: 1 10.1007/s12035-023-03842-6 [57]
IL-1β	F: TGCCACCTTTTGACAGTGATG<br>R: TGATGTGCTGCTGCGAGATT	138	60	doi: 10.1073/pnas.170549 1114 [58]

Abbreviations: AT, Anneaning temperture; GAPDH: Glyceraldehyde-3-phosphate dehydrogenase, ZO-1: zonula occludens-1, MUC2: , GPR41: G Protein-Coupled Receptor 41, GPR43: G Protein-Coupled Receptor 43, CYP3A11: Cytochrome P450 family 3 subfamily A member 11, TNF-α: Tumor Necrosis Factor Alpha,, IL-1β: Interleukin 1 Beta
